# The hemagglutinin proteins of clades 1 and 2.3.4.4b H5N1 highly pathogenic avian influenza viruses exhibit comparable attachment patterns to avian and mammalian tissues

**DOI:** 10.1128/jvi.00976-25

**Published:** 2025-09-23

**Authors:** Bingkuan Zhu, Kevin Fung, Hailey Huiyi Feng, Julia A. Beatty, Fraser Hill, Anne C. N. Tse, Christopher J. Brackman, Thomas H. C. Sit, Agnès Poujade, Nicolas Gaide, Mariette Ducatez, Gilles Foucras, Malik Peiris, Shih-Chieh Ti, John M. Nicholls, Hui-Ling Yen

**Affiliations:** 1 School of Public Health, LKS Faculty of Medicine, The University of Hong Kong 90397, Hong Kong SAR, China; 2 Department of Pathology, LKS Faculty of Medicine, The University of Hong Kong 570113, Hong Kong SAR, China; 3 Department of Veterinary Clinical Sciences, Jockey Club College of Veterinary Medicine and Life Sciences, City University of Hong Kong 597179, Hong Kong SAR, China; 4 CityU Veterinary Diagnostic Laboratory, City University of Hong Kong 53025, Hong Kong SAR, China; 5 Agriculture, Fisheries and Conservation Department, The Government of the Hong Kong SAR 258048 , Hong Kong SAR, China; 6 IHAP, Université de Toulouse, INRAE, Ecole Nationale Vétérinaire 137668, Toulouse, France; 7 School of Biomedical Sciences, LKS Faculty of Medicine, The University of Hong Kong 538789, Hong Kong SAR, China; University Medical Center Freiburg, Freiburg, Germany

**Keywords:** highly pathogenic avian influenza (HPAI), clade 2.3.4.4b, recombinant HA protein, tissue tropism

## Abstract

The global spread of the A/goose/Guangdong/1/96-lineage H5N1 highly pathogenic avian influenza (HPAI) viruses has been accompanied by an expanded host range and the establishment of sustained viral transmission among dairy cattle. To evaluate if the evolving H5N1 viruses have changed tissue tropism over time, we compared the binding patterns of recombinant hemagglutinin (HA) proteins derived from clade 1 (A/Vietnam/1203/04, H5VN) and circulating clade 2.3.4.4b viruses detected from wild bird (A/Eurasian Teal/Hong Kong/AFCD-HKU-23-14009-01020/2023, H5HK) and dairy cattle (A/bovine/Ohio/B24OSU-439/2024, H5OH). The HA protein of A(H1N1)pdm09 virus was included for comparison. Using bio-layer interferometry, H1 protein preferentially bound to the α2,6-linked sialoside 6′SLNLN, while H5 proteins preferentially bound to the α2,3-linked sialoside 3′SLN. H5OH showed higher binding affinity to 3′SLN than H5HK and H5VN. The attachment patterns of H1 and H5 proteins to the respiratory tissues of different species and dairy cattle mammary glands were evaluated. Compared to the H1 protein, H5 proteins showed stronger binding to the lung epithelial cells of cat, cattle, chicken, ferret, human, and pig, and the clade 2.3.4.4b H5 proteins exhibited increased binding to pig and cattle bronchial epithelial cells. All H5 proteins were attached to the alveolar and cistern epithelial cells in mammary glands, where α2,3-linked and α2,6-linked sialyl glycans were detected by *Maackia amurensis* lectin II and *Sambucus nigra* lectin, respectively. Taken together, the HA proteins of clade 1 and 2.3.4.4b H5N1 viruses generally share comparable attachment patterns to avian and mammalian tissues, despite evolving into antigenically distinct clades over the past 3 decades.

**IMPORTANCE:**

The outbreaks of H5N1 highly pathogenic avian influenza (HPAI) virus among US dairy cattle since 2024 have raised concerns of the potential changes in HA receptor binding specificity and tissue tropism. Using insect-cell-expressed recombinant HA proteins derived from clade 1 and circulating clade 2.3.4.4b H5N1 viruses, we showed that the dairy cattle H5 protein retained binding specificity for the avian-like α2,3-linked sialoside 3′SLN over the human-like α2,6-linked sialoside 6′SLNLN, with higher binding affinity to 3′SLN than the other H5 proteins. Clade 1 and 2.3.4.4b H5 proteins showed comparable attachment patterns to the mammary tissues of lactating dairy cattle, which showed high expression of α2,3-linked and α2,6-linked sialyl glycans. All H5 proteins also showed comparable attachment patterns to the lungs of cat, cattle, chicken, ferret, human, and pig. Our results suggest that the recent H5N1 outbreaks in dairy cattle may be related to ecological factors rather than changes in HA receptor binding specificity.

## INTRODUCTION

Since the emergence of the A/goose/Guangdong/1/96 (Gs/Gd) lineage of H5N1 highly pathogenic avian influenza virus (HPAI) from Southern China three decades ago, the hemagglutinin (HA) protein has evolved into multiple antigenically distinct clades, and the Gs/Gd-like viruses have spread across continents via migratory birds (1). The emergence and the expanded geographic distribution of clade 2.3.4.4b H5N1 viruses since 2020 has been accompanied by increasing numbers of outbreaks in domestic and wild bird species (2), spillover infections in humans and a wide range of mammalian species (3, 4), and the establishment of sustained viral transmission among dairy cattle, a new mammalian host for influenza A viruses (5–7).

Since March 2024, multiple states in the US have reported outbreaks of genotype B3.13 clade 2.3.4.4b H5N1 virus in dairy cattle (8). Infected cattle presented loss of appetite, massive drop in milk production, and mild respiratory signs (9). Field studies detected higher viral loads in the milk and mammary gland than those detected in the nasal swabs or lungs of infected dairy cattle (6). Experimental studies that used intra-nasal or intra-mammary gland inoculation routes also demonstrated preferential viral replication in the mammary glands over the respiratory tissues (9, 10). Importantly, dairy cattle with intra-mammalian inoculation of a genetically distinct clade 2.3.4.4b virus isolated from a wild goose in Europe (genotype euDG) showed comparable clinical signs as those inoculated with a genotype B3.13 dairy cattle isolate, suggesting that the ability to infect dairy cattle may be shared among clade 2.3.4.4b H5N1 viruses (9), a finding supported by the detection of genotype D1.1 of clade 2.3.4.4b in dairy cattle in January 2025 (11). These findings suggest that H5N1 outbreaks in dairy cattle may be attributed to both virological factors (e.g., ability to replicate in mammary gland tissue of dairy cattle) and ecological factors (e.g., shared milking devices and inter-state cattle movements). However, it is not clear if early Gs/Gd-lineage H5N1 viruses also possess the capacity to infect dairy cattle.

The receptor binding profile of HA proteins determines the host range and cell tropism of influenza A viruses. The HA proteins of avian influenza viruses preferentially bind to α2,3-linked sialyl glycans, while the HA proteins of human and swine influenza viruses preferentially bind to α2,6-linked sialyl glycans (2, 3). Here, using insect cell-expressed recombinant HA proteins, we compared the HA attachment pattern of clades 1 and 2.3.4.4b viruses to the mammary tissue of dairy cattle, as well as the respiratory tissues of cat, cattle, chicken, ferret, human, and pig.

## RESULTS

### Binding affinity of insect cell-expressed recombinant HA proteins to Neu5Acα2-3Galβ1-4GlcNAcβ-sp3-PAA-biot (3′SLN) and Neu5Acα2-6Galβ1-4GlcNAcβ1-3Galβ1-4GlcNAcβ-sp3-PAA-biot (6′SLNLN) by bio-layer interferometry assay

**Fig 1:**
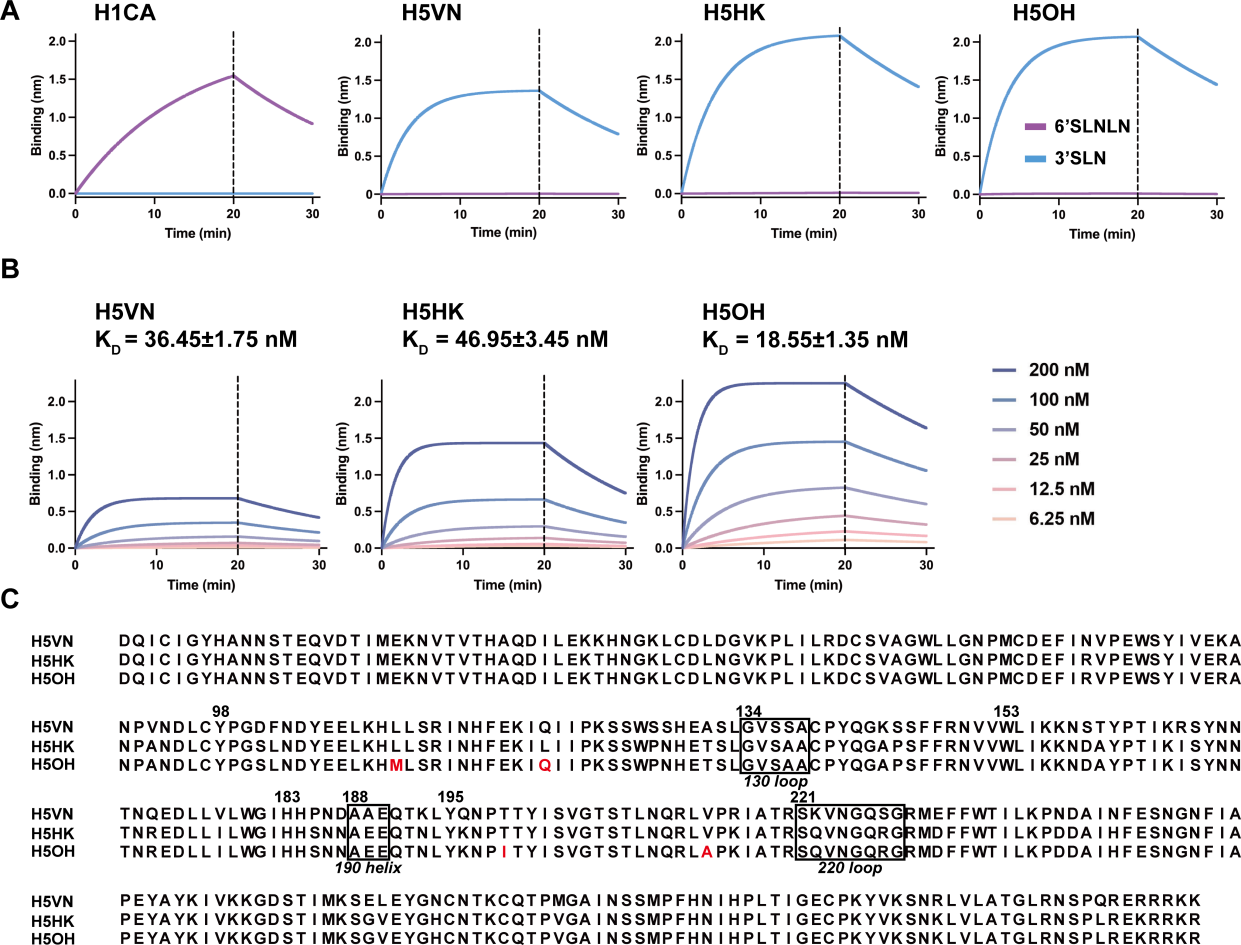
Binding kinetics of recombinant HA proteins to α2,3-linked and α2,6-linked sialyl glycans using BLI. (**A**) Streptavidin biosensors were immobilized with biotinylated α2,3-linked (3′SLN) or α2,6-linked (6′SLNLN) glycans at the concentration of 1 µg/mL, followed by incubation with recombinant HA proteins 67.5 nM for 20 minutes at 30°C. The binding signal over time is shown. (**B**) Kinetics of recombinant HA binding with 3′SLN measured by BLI. Curves were generated with equal densities of immobilized 3′SLN and the indicated purified HA protein concentrations. (**C**) Amino acid alignment of HA1 from H5 viruses in this study. Residues that differ between H5HK and H5OH were marked in red.

To compare the attachment pattern of HA protein of past and circulating H5N1 viruses, we expressed the HA proteins of A/Vietnam/1203/2004 (clade 1, abbreviated as H5VN, GISAID accession number EPI_ISL_21080, https://gisaid.org/), A/Eurasian Teal/Hong Kong/AFCD-HKU-23-14009-01020/2023 (clade 2.3.4.4b, abbreviated as H5HK, GISAID accession number EPI_ISL_19258154), and A/bovine/Ohio/B24OSU-439/2024 (clade 2.3.4.4b, abbreviated as H5OH, GISAID accession number EPI_ISL_19178083). For comparison, the HA protein from A/California/04/2009 A(H1N1)pmd09 virus (H1CA, GISAID accession number EPI_ISL_393964) was also expressed. The expressed HA proteins were evaluated for their binding affinity to 3′SLN and 6′SLNLN using bio-layer interferometry (BLI). All H5 proteins exhibited preferential binding to 3′SLN over 6′SLNLN, while the H1CA exhibited preferential binding to 6′SLNLN over 3′SLN (Fig. 1A). Further analysis using serially twofold diluted HA proteins (from 200 to 6.25 nM) to determine binding affinity, we observed that H5OH exhibited higher binding affinity (mean K_D_ ± SD = 18.55 ± 1.35 nM) than H5VN (36.45 ± 1.75 nM) and H5HK (46.95 ± 3.45 nM) (one-way analysis of variance [ANOVA], *P* < 0.01) (Fig. 1B). While the clade 1 H5VN differed by the clade 2.3.4.4b H5OH by 40 amino acids, the two clade 2.3.4.4.b H5HK and H5OH only differed by four amino acids (L111M, L122Q, T199I, V214A, H3 numbering) in HA1 (Fig. 1C). Specifically, the T199I change has been reported to increase the HA binding breadth to α2,3-linked *N-*acetyllactosamines of the first human H5N1 isolate (A/Texas/37/2024) reported in the dairy cattle outbreak (12).

### Clade 1 and clade 2.3.4.4b H5 proteins bind to the mammary tissues of lactating cows

**Fig 2:**
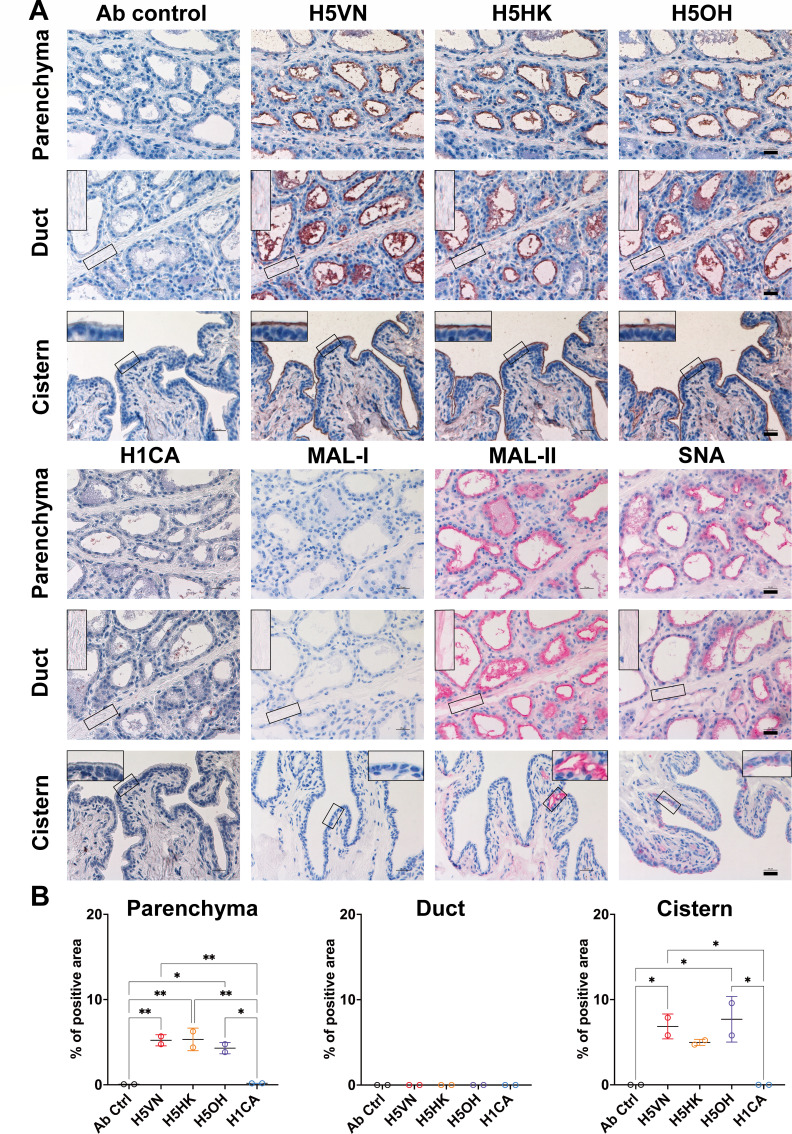
Attachment pattern of HA proteins and lectins to bovine mammary tissues. (**A**) Recombinant HA proteins (12.5 µg/mL) and plant lectins (20 µg/mL for SNA/MAL-I, and 10 µg/mL for MAL-II) were evaluated for their attachment pattern to formalin-fixed mammary gland tissues of lactating dairy cattle using protein histochemistry. The nuclei were counterstained with hematoxylin (blue). Inserts are digital magnifications of the boxed area. Scale bar indicates 25 µm. (**B**) The binding intensity of HA proteins was quantified using Qupath (version 0.5.1) from two independently repeated slides. The HA binding intensity to ductal and cistern epithelial cells was measured using the sub-panels showing the specific signals from the epithelial cells. One-way ANOVA was used to compare the binding intensity of different HA proteins. *, *P* < 0.05; **, *P* < 0.01.

Infection of mammary tissues has been a unique feature observed from the outbreaks of clade 2.3.4.4b H5N1 in dairy cattle (6, 9, 10). We compared the attachment pattern of H5VN, H5HK, H5OH, and H1CA to the mammary gland tissues of lactating cows (Fig. 2A). With recombinant proteins diluted to 12.5 µg/mL, we observed no binding of H1CA, but all three H5 recombinant proteins showed comparable binding intensity to the alveolar and cistern epithelial cells (Fig. 2B). It is noteworthy that no binding to the ductal epithelial cells was observed (Fig. 2B). To further characterize α2,3 and α2,6-linked sialyl glycans presented in the mammary tissues, we performed lectin staining with *Sambucus nigra* lectin (SNA) that preferentially binds to NeuAcα2,6Galβ1,4GlcNAc, *Maackia amurensis* lectin I (MAL-I) that preferentially binds to NeuAcα2,3Galβ1,4GlcNAc in N-glycans or O-glycans, and MAL-II that preferentially binds to NeuAcα2,3Galβ1,3GalNAc in O-glycans. Both SNA and MAL-II showed apparent binding to the alveolar epithelial cells and minor binding to the cistern epithelial cells. Interestingly, no apparent MAL-I binding was observed, suggesting that the mammary tissue of lactating cows may express low levels of NeuAcα2,3Galβ1,4GlcNAc (Fig. 2A).

### H5 proteins exhibited strong binding to lung epithelial cells of chicken, cat, cattle, ferret, human, and pig

**Fig 3:**
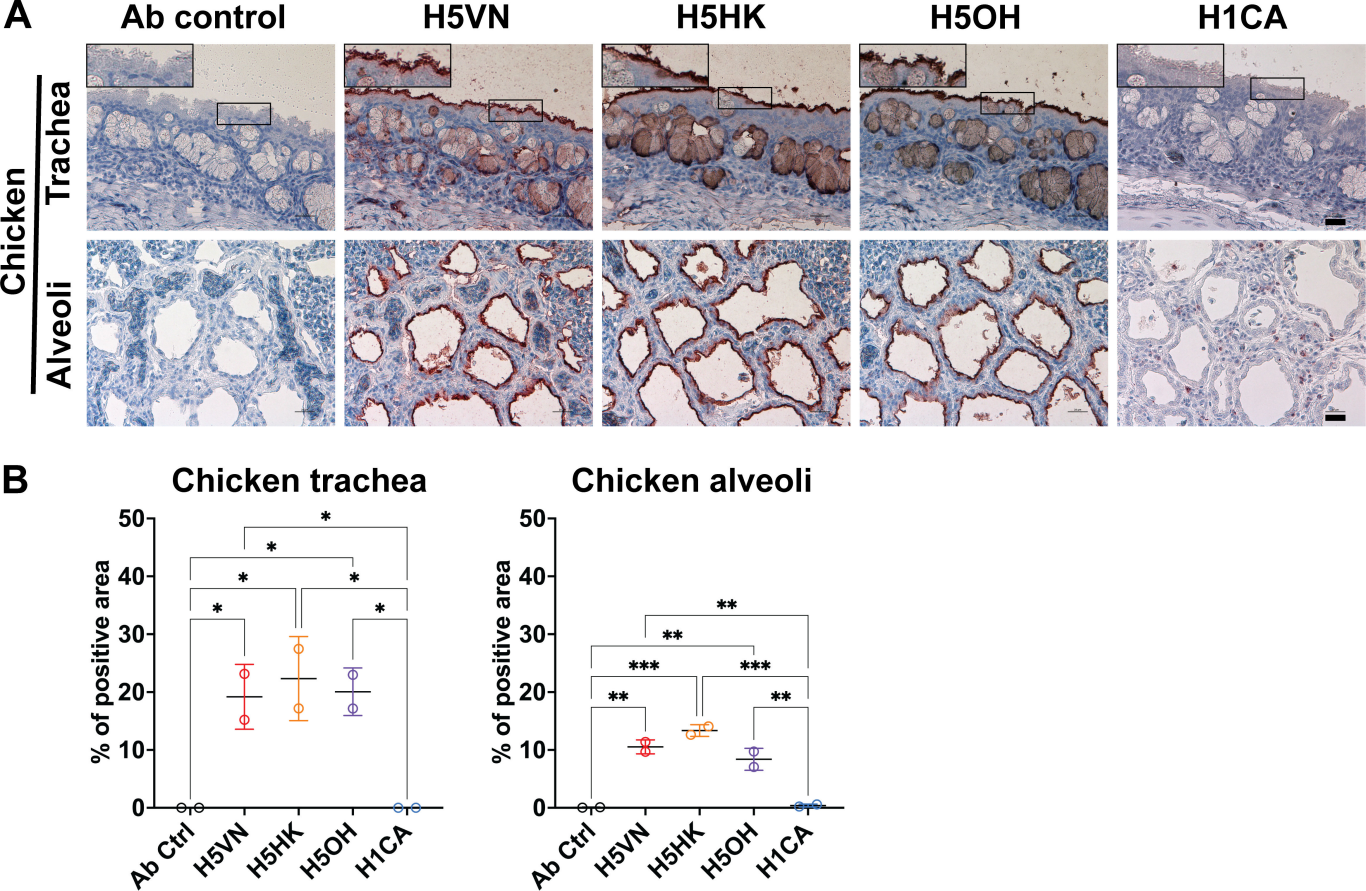
Attachment pattern of HA proteins to chicken respiratory tissues. (**A**) Recombinant HA proteins were diluted to 12.5 µg/mL and incubated with formalin-fixed chicken trachea and lungs using protein histochemistry. The nuclei were counterstained with hematoxylin (blue). Inserts are digital magnifications of the boxed area. Scale bar indicates 25 µm. (**B**) The binding intensity of HA proteins was quantified using Qupath (version 0.5.1) from two independently repeated slides. One-way ANOVA was used to compare the binding intensity of different HA proteins. *, *P* < 0.05; **, *P* < 0.01; ***, *P* < 0.001.

**Fig 4:**
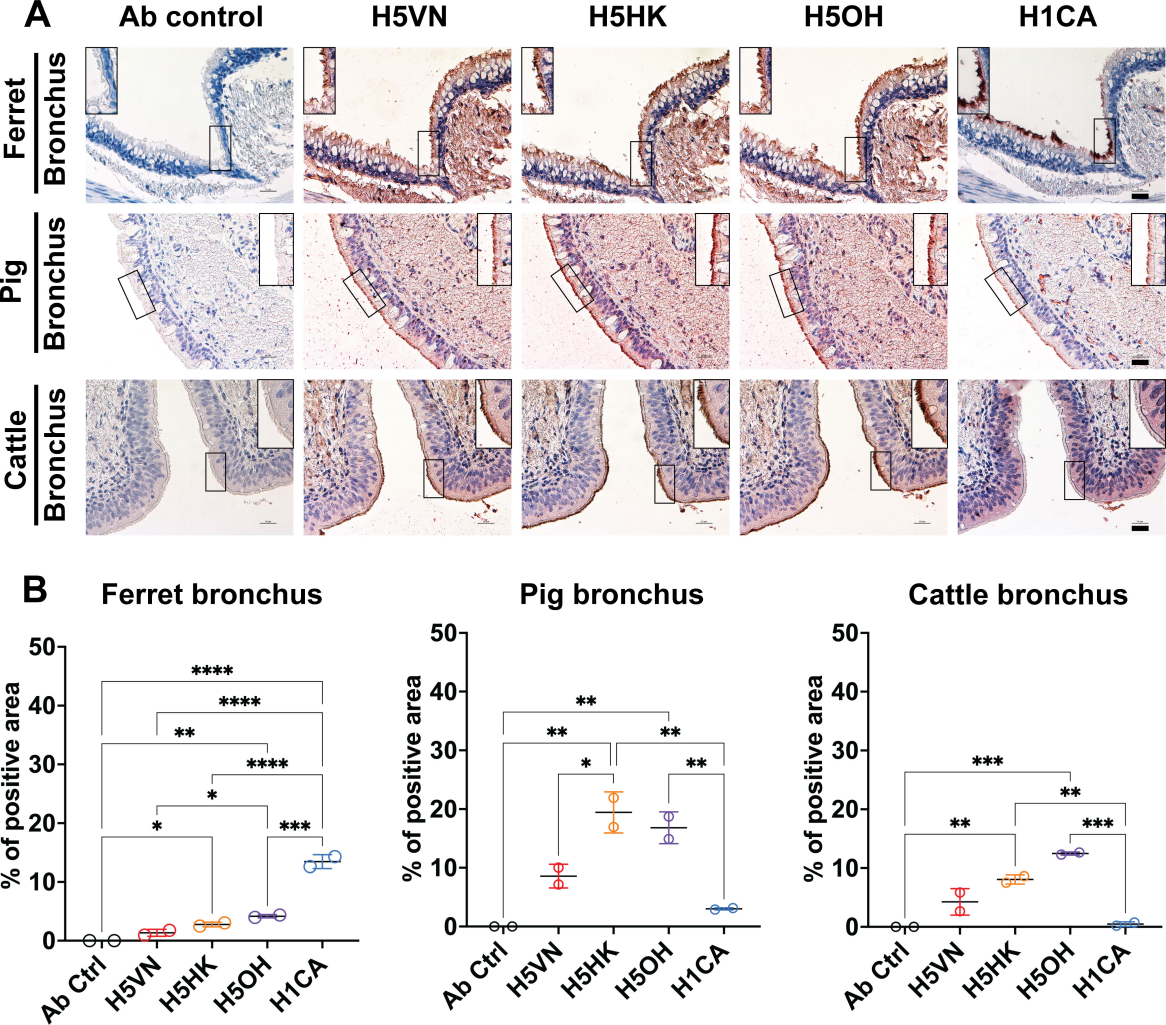
Attachment pattern of HA proteins to mammalian bronchial epithelial cells. (**A**) Recombinant HA proteins were diluted to 12.5 µg/mL and incubated with formalin-fixed bronchus from cattle, ferret, and pig using protein histochemistry. The nuclei were counterstained with hematoxylin (blue). Inserts are digital magnifications of the boxed area. Scale bar indicates 25 µm. (**B**) The binding intensity of HA proteins was quantified using Qupath (version 0.5.1) from two independently repeated slides, with measurements of the sub-panels showing the bronchial epithelial cells. One-way ANOVA was used to compare the binding intensity of different HA proteins. *, *P* < 0.05; **, *P* < 0.01; ***, *P* < 0.001; ****, *P* < 0.0001.

**Fig 5:**
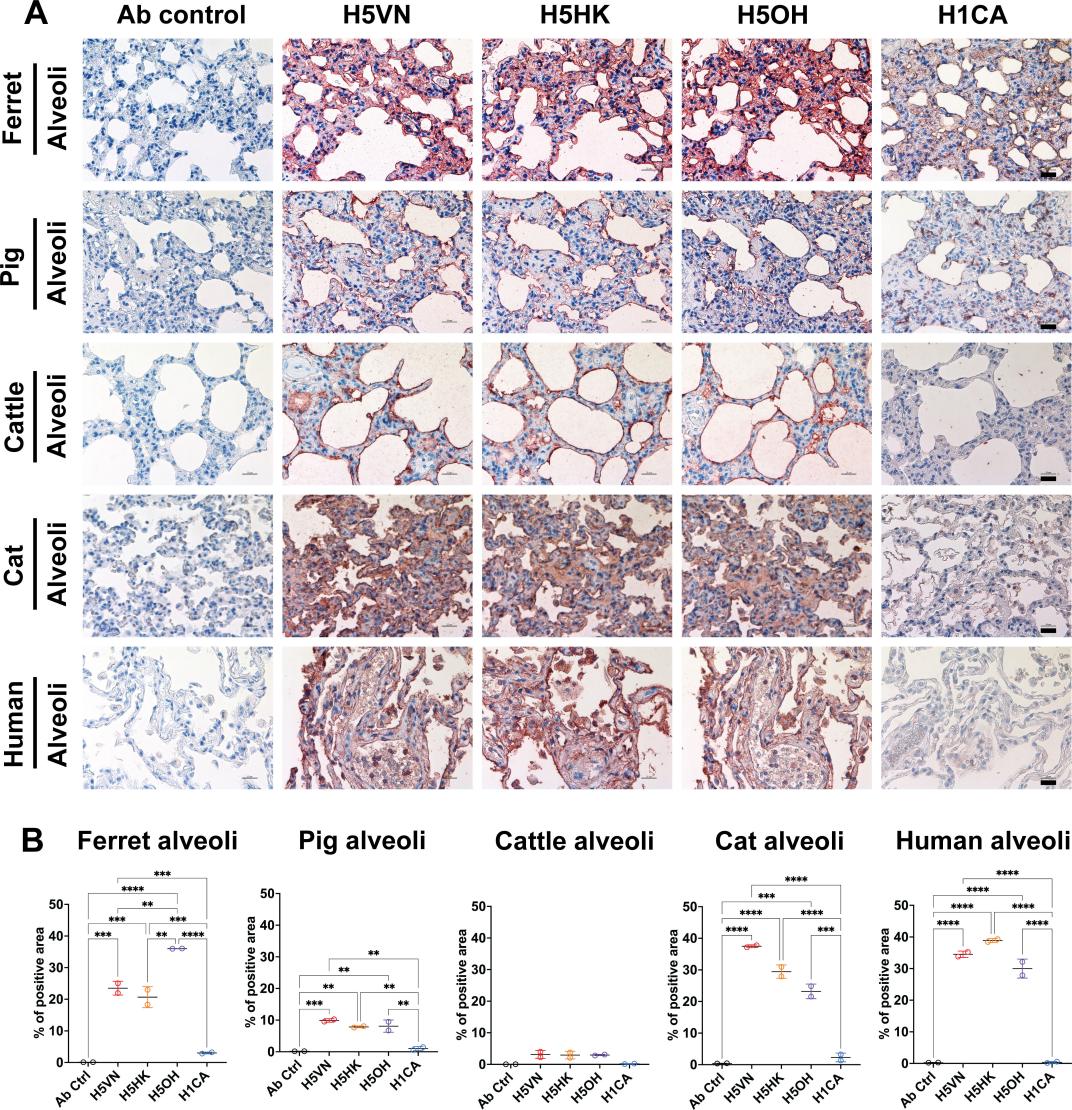
Attachment pattern of HA proteins to mammalian lung alveolar epithelial cells. (**A**) Recombinant HA proteins were diluted to 12.5 µg/mL and incubated to formalin-fixed lung tissues from cat, cattle, ferret, human, and pig using protein histochemistry. The nuclei were counterstained with hematoxylin (blue). Scale bar indicates 25 µm. (**B**) The binding intensity of HA proteins was quantified using Qupath (version 0.5.1) from two independently repeated slides. One-way ANOVA was used to compare the binding intensity of different HA proteins. **, *P* < 0.01; ***, *P* < 0.001; ****, *P* < 0.0001.

We further evaluated the attachment pattern of recombinant HA proteins to the respiratory tissues of different species. All H5 protein binds to chicken trachea and lung epithelial cells, while no binding of H1CA was observed (Fig. 3A and B). The recombinant HA proteins also exhibited different attachment patterns to the mammalian respiratory tissues (Fig. 4 and 5). In the ferret bronchus, H1CA showed patchy (Fig. 4A) but a higher overall binding signal (Fig. 4B) to the bronchial epithelial cells than the H5 proteins, while the clade 2.3.4.4b H5 proteins showed stronger binding to pig and cattle bronchus than H1CA (Fig. 4A and B). All three H5 recombinant proteins showed stronger binding to the lung epithelial cells of cat, cattle, human, and pig than the H1 protein, with the H5OH exhibiting stronger binding to the ferret lung epithelial cells than H5HK or H5VN (Fig. 5A and B). Previous studies have reported that H5N1 and avian influenza viruses generally showed stronger attachment to lung tissues of various species than the human seasonal influenza viruses (13, 14). Taken together, the results suggest that the HA proteins derived from clade 1 and clade 2.3.4.4b H5N1 viruses showed minor differences in their attachment patterns to the respiratory tissues of different species.

## DISCUSSION

The expanded host range and the sustained transmission of clade 2.3.4.4b H5N1 viruses among dairy cattle have raised concerns about the potential changes in the HA attachment pattern to sialyl glycans expressed on animal tissues, which may affect viral transmissibility and pandemic potential. Previous studies have shown that dairy cattle viruses exhibited preferential binding for α2-3-linked sialosides and tissue tropism that resembles avian influenza viruses, including early Gs/GD-lineage H5Nx strains (7, 12, 15–23). Similar to our study design, two previous studies used mammalian (18) or insect-cell expressed (7) recombinant HA proteins derived from clade 1, clade 2.1.2.3, and 2.3.4.4b H5Nx viruses to evaluate HA attachment pattern to bovine (trachea, bronchioles, lungs, mammary gland, conjunctiva), equine (trachea and lungs), porcine (trachea and lungs) and human (trachea, bronchioles, lungs, mammary gland, conjunctiva) tissues, with similar findings. While our results are consistent with these two studies, we additionally showed the binding pattern of clade 1 and clade 2.3.4.4b H5 proteins to the lung tissue of cat and ferret, as well as the bronchus tissue of cattle, ferret, and pig. We also observed that the clade 2.3.4.4b H5 proteins exhibited increased binding to pig and cattle bronchial epithelial cells than the H1 protein, suggesting that pigs may be susceptible to infection of clade 2.3.4.4b viruses (24), and further co-infection with other swine influenza viruses may pose a risk for the emergence of novel reassortant viruses. Considering available literature to date, the results suggest the H5 proteins derived from the past and circulating clade 2.3.4.4b H5Nx viruses generally share a comparable attachment pattern to the cattle mammary tissues and the respiratory tissues of different hosts. This suggests that the H5N1 outbreaks in dairy cattle may be related to ecological factors rather than changes in HA receptor binding specificity. Additional epidemiological studies and environmental sampling are needed to identify risk factors associated with the introduction of H5N1 into dairy herds.

Using BLI, we observed that all H5 proteins preferentially bind to 3′SLN with no detectable binding to 6′SLNLN. H5OH also showed higher binding affinity to 3′SLN than H5HK or HKVN. The BLI assay is sensitive and quantitative, but only allows evaluating HA binding to a specific glycan at a time. On the other hand, the use of recombinant HA proteins allowed assessing viral tropism in tissues presented with diverse glycan structures, although this method is more qualitative than quantitative. It is interesting to note that while H5VN showed lower binding to 3′SLN than H5OH, H5VN appeared to show similar attachment pattern as H5OH to various animal tissues. In agreement with our finding, glycan array analysis (16) showed H5VN demonstrated reduced binding to 3′SLN than the clade 2.3.4.4b A/Texas/37/2024 virus (with identical HA sequence as H5OH). Another study also showed that the H5VN exhibited different binding patterns than the HA of A/bovine/Ohio/B24OSU-432/2024 (with identical HA sequence as H5OH) to bi-antennary N-glycans and O-glycans (19). Collectively, these findings demonstrated the strength of different experimental methods and the need to consider different platforms while assessing HA binding specificity and tissue tropism.

Experimental infection showed that the mammary gland may serve as the main site of replication in dairy cattle by the clade 2.3.4.4b H5N1 viruses (9, 10). Since clade 1 and clade 2.3.4.4b H5 proteins all bind to the alveolar and cistern epithelial cells in the mammary glands, it is likely that clade 1 H5N1 virus may similarly cause infection in mammary gland tissue, given the proper opportunity. The attachment pattern of H5 proteins in mammary glands is in accordance with the results reported by previous studies (7, 18). Using lectin staining, we observed predominant expression of NeuAcα2,6Galβ1,4GlcNAc (detected by SNA) and NeuAcα2,3Galβ1,3GalNAc (detected by MAL-II) in the mammary gland tissues of lactating dairy cattle, while the expression of NeuAcα2,3Galβ1,4GlcNAc (detected by MAL-I) was low. This result, in combination with the H5 protein attachment pattern, suggests H5 proteins bind to the NeuAcα2,3Galβ1,3GalNAc O-glycans in the mammary tissues (12). Interestingly, although α2,6-linked sialyl glycans were distributed along the glandular alveolar and cistern epithelial cells, we did not observe any binding of the H1 protein to these tissues. Our result is consistent with those reported previously, including limited attachment of recombinant H1 protein derived from a mouse-adapted influenza strain A/Puerto Rico/8/34 to dairy cattle mammary tissues (18), and restricted replication of an A(H1N1)pdm09 virus in the *ex vivo* culture of bovine mammary gland (25).

The major limitation of our study is that the HA attachment pattern alone is insufficient to infer viral replication efficiency in these animal tissues, as viral replication is also determined by the viral gene constellation and host-adaptive amino acid changes. As the circulating clade 2.3.4.4b viruses continue to expand their genetic diversity through frequent genetic reassortments with other avian influenza viruses (26–29), the replication efficiency of the genetically diverse clade 2.3.4.4b viruses in different host species needs to be further verified experimentally. With comparable viral polymerase activity, Bauer et al. (30) demonstrated that a clade 2.3.4.4b virus showed better attachment and replicated more efficiently than a clade 2.1.3.2 virus in human nasal and tracheal/bronchiolar epithelial cells. Using recombinant HA proteins, Carrasco et al. (31) and Song et al. (7) also reported clade 2.3.4.4b virus attached better to the human tracheal epithelial cells than the clade 2.1.3.2 virus.

Taken together, the data available to date support that the clade 2.3.4.4b retained a comparable receptor binding profile as the early H5N1 viruses. However, it’s important to note that H5N1 viruses continue to cause spillover infections in mammals, which provide opportunities for viral adaptation (32). A study reported that a single Gln226Leu mutation may switch the cattle H5N1 virus binding specificity to human-type receptors (15). Continuous efforts on surveillance and monitoring of the evolution of H5N1 viruses isolated from different host species are essential for pandemic preparedness.

## MATERIALS AND METHODS

### Expression of recombinant HA proteins

Soluble recombinant HA proteins were expressed and purified following established protocols (33, 34). Genes encoding the ectodomains of the HA protein of the A(H1N1)pdm09 and A(H5N1) (with the multibasic cleavage site removed) viruses were subcloned into the baculovirus transfer vector pFastBac1 (Invitrogen), in frame with an N-terminal gp67 signal peptide, a C-terminal trimerization foldon sequence from bacteriophage T4, followed by a thrombin cleavage site and a His_6_-tag. Transfection and baculovirus amplification were performed in Sf9 cells using the Bac-to-Bac baculovirus expression system (Invitrogen). On day 3 post-infection, supernatants were harvested, and the HA proteins were purified using Ni-charged immobilized metal affinity chromatography resin (Bio-Rad). Recombinant proteins were stored in PBS with 20% sucrose (Sigma) in aliquots at −80°C.

### HA binding affinity to glycans using bio-layer interferometry

The expressed recombinant HA proteins were evaluated for their binding affinity to biotinylated 3′SLN (Neu5Acα2-3Galβ1-4GlcNAcβ-sp3-PAA-biot) (GlycoNZ) and biotinylated 6′SLNLN (Neu5Acα2-6Galβ1-4GlcNAcβ1-3Galβ1-4GlcNAcβ-sp3-PAA-biot) (GlycoNZ) using BLI. Assays were performed using the Octet Red96e system (FortéBio) in 96-well microplates as previously described (35). Dulbecco’s PBS with calcium, magnesium, and 0.005% Tween-20 was used as the assay buffer to reconstitute protein and glycan molecules. Biotinylated 3′SLN and 6′SLNLN (GlycoNZ) were preloaded to the streptavidin-coated biosensors (FortéBio) at 1 µg/mL for 10 min. HA proteins diluted to 67.5 nM were pre-conjugated with the mouse anti-His-tag antibody (Thermo Scientific, clone # MA1-21315) and the horseradish peroxidase (HRP)-conjugated goat anti-mouse secondary antibody (Abcam, clone # ab6789) at the molar ratio of 2:1:2 for 30 min on ice before being added to the wells. The 96-well plates were incubated at 30°C for 30 min, with sample plates agitated at 1,000 RPM. Binding kinetics of H5 proteins to 3′SLN were measured for 20 min at HA concentrations of 6.25, 12.5, 25, 50, 100, and 200 nM. The data were fitted with a 1:1 binding model to evaluate HA binding affinity (K_D_) to 3′SLN.

### Immunohistochemical staining with recombinant HA proteins

Formalin-fixed and paraffin-embedded tissue blocks and sections were prepared by the Department of Pathology, Li Ka Shing Faculty of Medicine, The University of Hong Kong or obtained from collaborating laboratories. Immunohistochemistry was performed according to a previously described protocol (18) with minor modifications. Briefly, the sections were dried at 60°C for 20 min, deparaffinized, and rehydrated. Antigen retrieval was achieved by boiling the sections in 10 mM sodium citrate (pH 6.0) for 20 min. Endogenous peroxidase activity was quenched using 3% hydrogen peroxide, and nonspecific binding was blocked with 10% goat serum (Giobco). Histidine-tagged recombinant HA proteins were diluted to 12.5 µg/mL and were pre-conjugated with the mouse anti-His-tag primary antibody and the HRP-conjugated goat anti-mouse secondary antibody (Abcam) as described. This pre-complexed mixture was then applied to the tissue sections and incubated for 2 h at room temperature, followed by washing with PBS buffer containing 0.05% (vol/vol) Tween-20 (PBST). The chromogen 3-amino-9-ethylcarbazole (AEC) (Sigma-Aldrich) was used as the HRP substrate. Tissues were counterstained with Gill’s hematoxylin (Vector Laboratories), mounted with permanent aqueous mounting medium (Bio-Rad), and examined using a Nikon Eclipse Ti-S microscope. The experiments were repeated twice independently.

### Lectin staining

To evaluate the distribution of α2,3-linked and α2,6-linked sialyl glycans in tissue slides, biotinylated *Sambucus nigra* lectin (SNA), *Maackia amurensis* lectin I (MAL-I), and *Maackia amurensis* lectin II (MAL-II) were used for staining. SNA is known to preferentially bind to α2,6-linked terminal sialic acids (SA) (Neu5Acα2,6Galβ1,4GlcNAc). MAL-I and MAL-II preferentially bind to α2,3-linked terminal SA, but MAL-I preferred Neu5Acα2,3Galβ1,4GlcNAc while MAL-II preferred Neu5Acα2,3Galβ1,3GalNAc. Antigen retrieval and endogenous peroxidase blocking were performed as described above. After blocking with 0.1% bovine serum albumin (Sigma-Aldrich), the slides were incubated with 20 µg/mL SNA/MAL-I, or 10 µg/mL MAL-II at room temperature for 1 h, followed by incubation with alkaline phosphatase-conjugated streptavidin (Vector Laboratories) for 45 min. The slides were then developed with Vector Red Substrate Kit (Vector Laboratories). After counterstaining with Gill’s hematoxylin (Vector Laboratories), the slides were counterstained with Scott’s tap water (Sigma-Aldrich), air-dried, and mounted with Permount (Fisher Scientific).

### Statistical analysis

To compare HA binding intensity to the respiratory and mammary tissues, the chromagen AEC signal was quantified using the Qupath software (36) from two independently stained tissue slides, as shown. A one-way ANOVA test was used to compare the binding signal of different recombinant HA proteins.

## Data Availability

The authors confirm that the data supporting the findings of this study are available within the article.
